# Multiple insecticide resistance target sites in adult field strains of *An.gambiae* (*s.l*.) from southeastern Senegal

**DOI:** 10.1186/s13071-020-04437-z

**Published:** 2020-11-11

**Authors:** El hadji Diouf, El hadji Amadou Niang, Badara Samb, Cheikh Tidiane Diagne, Mbaye Diouf, Abdoulaye Konaté, Ibrahima Dia, Ousmane Faye, Lassana Konaté

**Affiliations:** 1 Laboratoire d’Écologie Vectorielle et Parasitaire, Université Cheikh Anta Diop de Dakar, Dakar, Senegal; 2 Institut Pasteur de Dakar, Dakar, Senegal

**Keywords:** Insecticide resistance, *An.arabiensis*, *An.coluzzii*, *An.gambiae (s.s.)*, *Vgsc*-*1014F*, *Vgsc-1014S*, *Ace-1*, A296S, A296G, Senegal

## Abstract

**Background:**

High coverage of long-lasting insecticidal nets (LLINs) and indoor residual spraying (IRS) are the cornerstones of vector control strategy in Senegal where insecticide resistance by the target vectors species is a great of concern. This study explores insecticide susceptibility profile and target-site mutations mechanisms within the *Anopheles* *gambiae* complex in southeastern Senegal.

**Methods:**

Larvae of *Anopheles* spp. were collected in two sites from southeastern Senegal Kedougou and Wassadou/Badi in October and November 2014, and reared until adult emergence. Wild F_0_ adult mosquitoes were morphologically identified to species. Susceptibility of 3–5-day-old *An*. *gambiae* (*s.l.*) samples to 11 insecticides belonging to the four insecticide classes was assessed using the WHO insecticide susceptibility bioassays. Tested samples were identified using molecular techniques and insecticide resistance target-site mutations (*kdr*, *ace-1* and *rdl*) were determined.

**Results:**

A total of 3742 *An.* *gambiae* (*s.l*.) were exposed to insecticides (2439 from Kedougou and 1303 from Wassadou-Badi). Tests with pyrethroid insecticides and DDT showed high level of resistance in both Kedougou and Wassadou/Badi. Resistance to pirimiphos-methyl and malathion was not detected while resistance to bendoicarb and fenitrothion was confirmed in Kedougou. Of the 745 specimens of *An.* *gambiae* (*s.l*.) genotyped, *An.* *gambiae* (*s.s*.) (71.6%) was the predominant species, followed by *An.* *arabiensis* (21.7%), *An.* *coluzzii* (6.3%) and hybrids (*An*. *gambiae* (*s.s*.)*/An.* *coluzzii*; 0.4%). All target site mutations investigated (*Vgsc*-1014F, *Vgsc*-1014S, *Ace-1* and *Rdl*) were found at different frequencies in the species of the *Anopheles* *gambiae* complex. *Vgsc*-1014F mutation was more frequent in *An.* *gambiae* (*s.s*.) and *An.* *coluzzii* than *An.* *arabiensis*. *Vgsc*-1014S was present in *An.* *gambiae* (*s.l*.) populations in Wassadou but not in Kedougou. *Ace-1* and *rdl* mutations were more frequent in *An.* *gambiae* (*s.s*.) in comparison to *An.* *arabiensis* and *An.* *coluzzii*.

**Conclusions:**

Resistance to all the four insecticide classes tested was detected in southeastern Senegal as well as all target site mutations investigated were found. Data will be used by the national Malaria Control Programme.
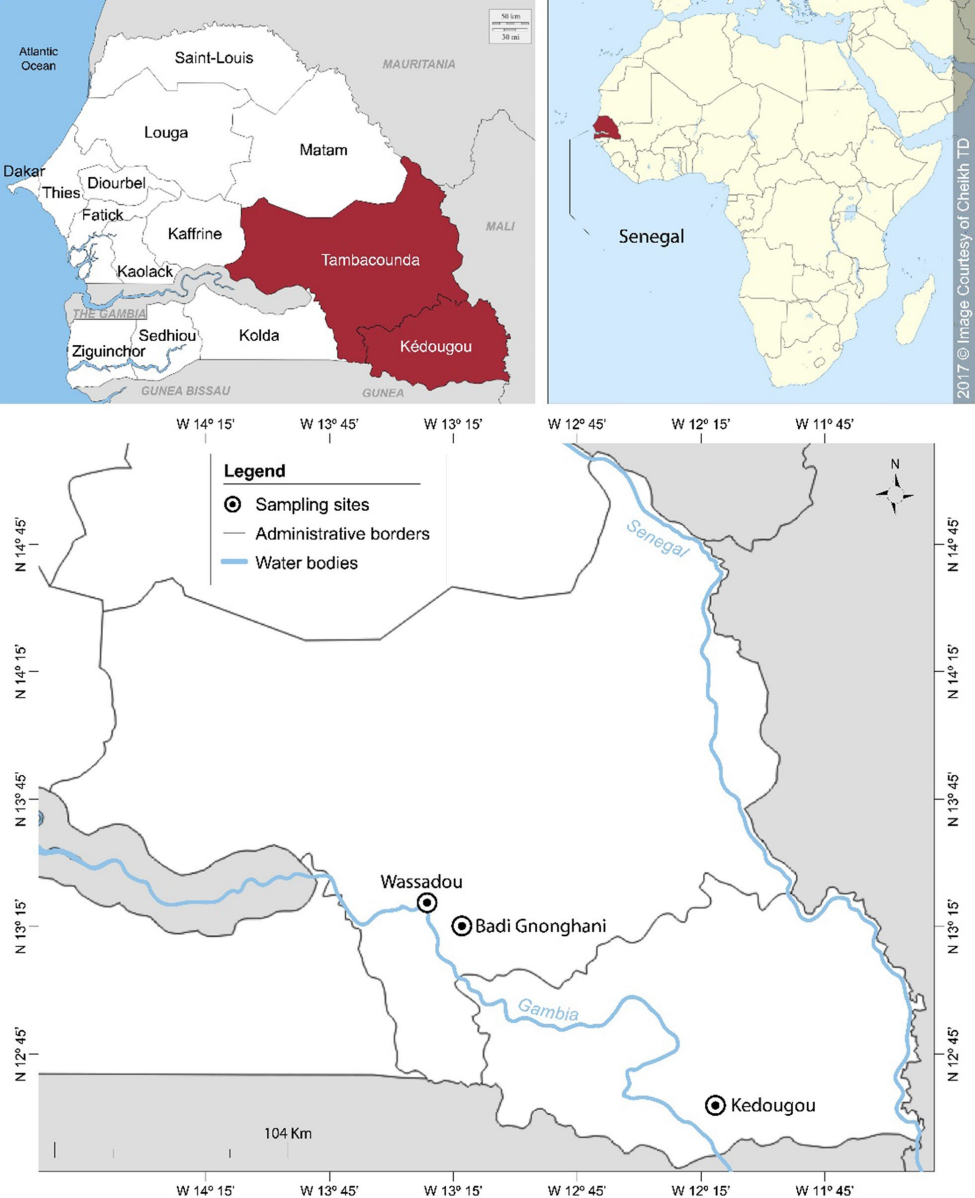

## Background

Malaria remains a major public health challenge in endemic countries. It mainly affects vulnerable groups, including pregnant women and children less than five years-old. In most endemic countries, the fight against this endemic disease is based on (i) the early detection of *Plasmodium* infection by a biological diagnosis of cases (rapid diagnosis test and blood smear), (ii) treatment with effective drugs (artemisinin-based combination therapy (ACT)), and (iii) prevention (intermittent preventive treatment in pregnancy, seasonal malaria chemoprevention in children under ten years-old and vector control). Worldwide, the number of malaria cases has decreased from 251 million in 2010 to 228 million in 2018 At the same time, the disease incidence declined from 71 to 57 cases per 1000 in 2010 and 2018, respectively [1].

Despite these advances, malaria incidence in Africa has increased between 2014 and 2016 [2], because of factors including the development and spread of insecticide resistance in the main malaria vectors, such as *An.* *funestus* and *An.* *gambiae* (*s.l*.). Currently, resistance to at least one of the four major classes of insecticides has been reported in malaria vectors in all African endemic areas [1]. This could be a major obstacle to the efficacy of insecticide-based vector control strategies [3, 4]. Resistance to pyrethroids (the only insecticide class currently approved for long-lasting insecticidal mosquito nets: LLINs) [3, 5, 6] and to DDT (dichlorodiphenyltrichloroethane) [7] has been reported in many endemic settings [8, 9], particularly in Asia and in tropical African countries.

Several insecticide resistance mechanisms have been described in the major malaria vectors. The “knock down” resistance (*kdr*) mutation, which confers resistance to pyrethroids and DDT, is the most common. It occurs at 1014 position of the gene encoding the S6 Trans membrane domain II of para voltage-gated sodium channel (*Vgsc*), the interaction site of insecticides and protein targets.

Two types of *kdr* mutation, widely distributed in species of the *An.* *gambiae* complex, are reported in many studies in Africa [10–12]. Both mutations are due to substitution: the first mutation changes a leucine to a phenylalanine at amino acid position 1014 of voltage gated sodium channel gene (*Vgsc*-1014F), whereas the second mutation changes a leucine to a serine at amino acid position 1014 of the same gene: (*Vgsc-*1014S) [13, 14].

Other target-site mutations have been described in *An.* *gambiae* (*s.l*.). These include the gene encoding acetyl cholinesterase (*ace-1*) and the gamma-amino butyric acid (GABA) receptor [15]. The *ace-1*^*R*^ (acetyl cholinesterase insensible) mutation, caused by a substitution of a glycine to a serine at position 119 (G119S), results in insensitivity of acetyl cholinesterase (AChE1) to organophosphates and carbamates [16]. The GABA receptor mutation results from a nucleotide substitution at amino acid position 296, leading the change of an alanine to a serine in *An.* *arabiensis* or a glycine in *An.* *gambiae* (*s.s.*) and *An.* *coluzzii*. It generally confers resistance to dieldrin (*rdl*-A296S or *rdl-*A296G) or can lead to a cross-resistance to cyclodiene organochlorines and phenyl pyrazole (fipronil) [17].

Previous studies of susceptibility of *An.* *gambiae* (*s.l*.) to insecticides have revealed, often at different levels, a phenotypic resistance to DDT and to pyrethroids in most parts of Senegal, except in the north and extreme southeast [18–20]. The aims of this study were to update the current status of insecticide resistance among *An.* *gambiae* (*s.l*.) populations in southeastern Senegal and to identify the mechanisms of insecticide resistance, particularly for target-site mutations involved in insecticide resistance.

## Methods

### Study area

**Fig. 1:**
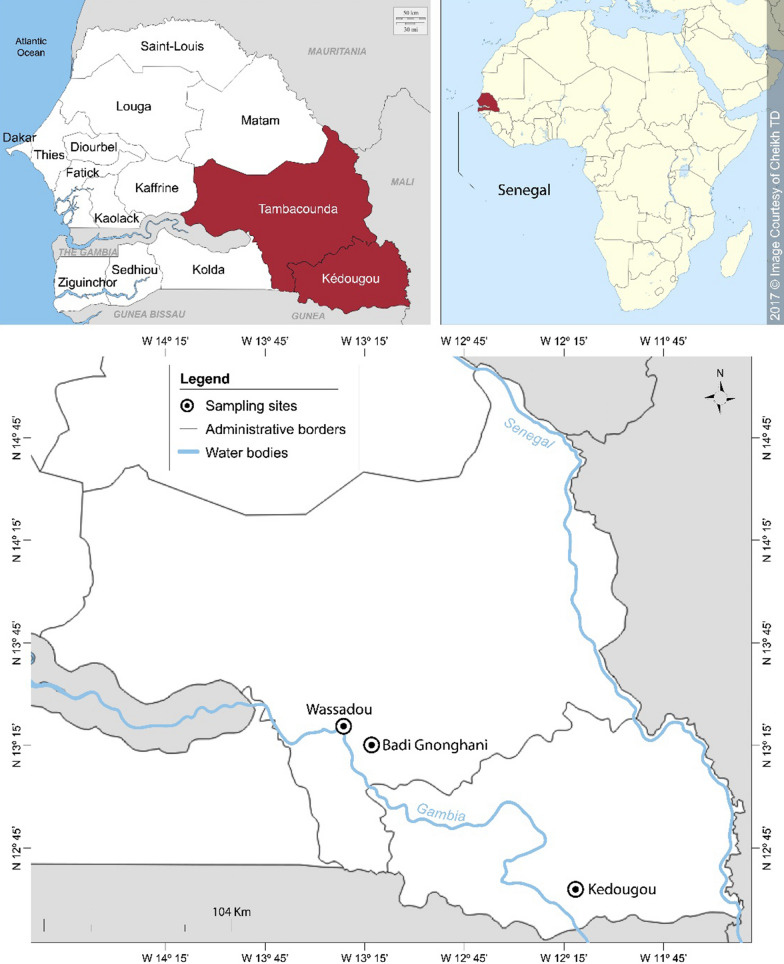
Map showing mosquito sampling areas in southeastern Senegal

The study was conducted in October and November 2014 in two sites of southeastern Senegal: Kedougou (12°33ʹ11.3ʺN, 12°10ʹ 09.5ʺW) in Kedougou district and Wassadou-Badi (13°22ʹ 22.3ʺN, 13°22ʹ 53.5ʺW) in Tambacounda district (Fig. 1). The area is bordered by the Republics of Mali and Guinea. The climate is a Sudano-Guinean type with a rainy season generally extending from May to October [21]. The average precipitation is between 1200–1300 mm per year with average temperatures between 33–42 °C for maxima and 21–25 °C for minima. Agriculture is the main economic activity with a wide production of sorghum, maize, fonio, rice and cotton. The area of Kedougou is also a gold-mining zone, and has a significant potential for mineral resources. With a malaria incidence greater than 25 per 1000 [22], the study area remains the most holo endemic area in Senegal. In 2014, 265,624 clinical cases were recorded, including 12,636 severe cases [23]. Malaria transmission is seasonal and occurs during the rainy season and the beginning of the dry season. *Anopheles* *gambiae* (*s.s*.), *An.* *coluzzii* and *An.* *arabiensis* are responsible for most malaria transmission, but in some specific settings, *An.* *funestus* and *An.* *nili* are involved [24, 25].

### Collection of immature stages of *Anopheles* spp. and mosquito rearing

Larval collections were carried out in Kedougou, Wassadou and Badi. Wassadou and Badi belong to the same area and are just 1.5 km apart. The larval sites for *An.* *gambiae* (*s.l*.) consisted of temporary water collections, footprints and hollows associated with human activities. During the study period, immature stages were collected from positive larval sites located in or around villages. All larval collections from Kedougou were pooled to form a sample and those of Wassadou and Badi a sample. After collection, immature stages were transferred to a local insectary for rearing. *Anopheles* larvae were fed with fishmeal (Tetramin Baby^®^; tetraGmbH, Herrenteich 78, Germany). Pupae were collected daily and introduced into rearing cages. At emergence, mosquito adults were fed using absorbent cotton soaked with 10% sucrose solution.

### WHO bioassay tests and morphological identification

WHO susceptibility tests were performed according to the standardized protocol [26] with adults 3 to 5 days post-emergence from field collected larvae. Eleven insecticides belonging to four insecticide classes were tested: five pyrethroids (0.05% deltamethrin, 0.75% permethrin, 0.05% lambda-cyhalothrin, 0.1% alpha-cypermethrin and 0.15% cyfluthrin), two organochlorines (4% DDT and 4% dieldrin), three organophosphates (1% fenitrothion, 5% malathion and 1% pirimiphos-methyl) and one carbamate (0.1% bendiocarb). For the pyrethroids and DDT, the number of knockdown individuals was recorded at 10, 15, 20, 30, 40, 50 and 60 min during the exposure period. Mortality rates were determined 24 h post-exposure. The mortality rates in the tested groups were corrected when needed, using Abbot’s formula [27] to validate tests results according to mortality rate in controls.

Finally, tested specimens were identified morphologically under a binocular microscope using a conventional key [28] and then individually stored in Eppendorf tubes containing silica-gel. Surviving specimens and 110 randomly selected dead specimens (10 for bendiocarb, 20 for organochlorines, 30 for organophosphates and 50 for pyrethroids) were individually stored for laboratory analysis.

### DNA extraction, molecular identification of species and detection of *kdr*, *ace-1* and *rdl*

Genomic DNA extraction was carried out by the 2% CTAB (cetyl trimethyl ammonium bromide) method [29] adapted to animal tissues. Each sample was grounded in an Eppendorf tube containing 200 μl of CTAB and incubated at 65 °C for 1 h. Then 200 μl of chloroform was added and mixed by inversion. The mixture was centrifuged at 12,000× *rpm* for 5 min, after which the supernatant containing DNA was recovered in a new Eppendorf tube. DNA was then precipitated with isopropanol and the mixture was then centrifuged at 12,000× *rpm* for 15 min and washed with 70% ethanol after a centrifugation of 12,000× *rpm* for 5 min and then brought to speed-vac for drying. DNA was suspended in molecular biology grade water: DNA/RNA free (Cat # 10977 035; Invitrogen, Grand Island, NY, USA). One tenth of dilution was carried out before PCR (identification of species of the *An*. *gambiae* complex and detection of target site mutations). Species were identified using IMP-PCR (intentional mismatch primer-PCR) as described by Wilkins et al. [30]. *Kdr* mutations (*Vgsc*-1014F and *Vgsc*-1014S), G119S (*ace-1*^*R*^) and *rdl-*296S (*An.* *arabiensis*) and *rdl-*296G (*An.* *gambiae* (*s.s.*) and *An.* *coluzzii*) were determined using the protocols described by Huynh et al. [31] and by Weill et al. [32] and Du et al. [17] respectively.

### Data entry and statistical analysis

Data were recorded in a Microsoft Excel 2010 spreadsheet. Homogeneity tests of percentages and averages were performed using standard chi-square tests with a 5% significance level threshold. The level of insecticide susceptibility of mosquitoes was evaluated following the WHO criteria [26] and validated by considering mortality rates of control mosquitoes. If the control mortality was less than 5%, no correction of test results was necessary whereas mortality of ≥ 5% required Abbott’s correction [26]. KDT_50_ and KDT_95_ times with 95% confidence intervals were determined using a log-probit regression model. The mortality rates, the genotypes and allelic frequencies were estimated for each studied population. All statistical analyses and graphs were made using R software version 3.0.3 [33].

## Results

### Susceptibility tests

**Fig. 2:**
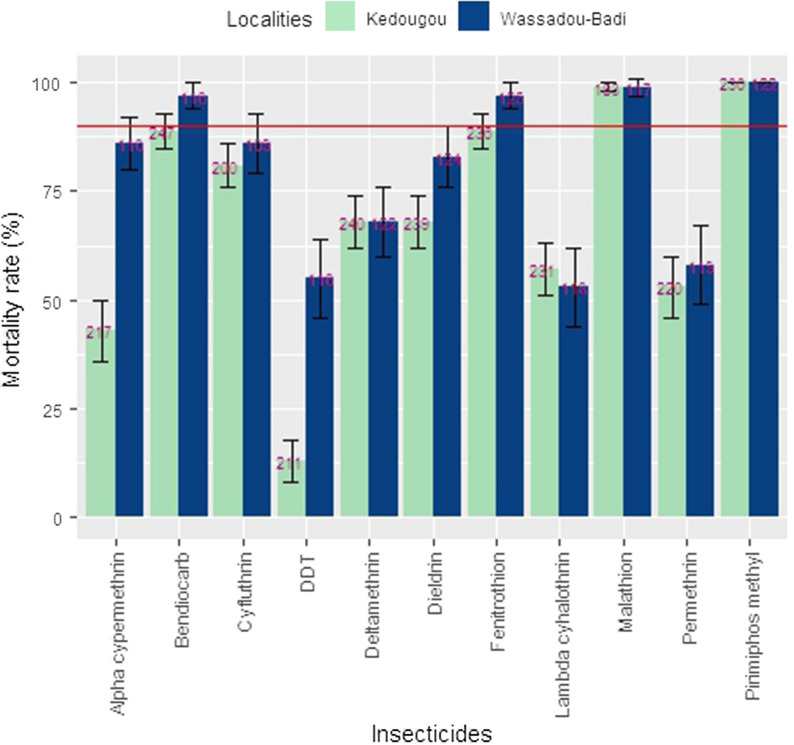
Mortality rates of *Anopheles gambiae* (*s.l*.) 24 h populations after exposure to WHO recommended insecticide doses in October and November 2014

A total of 3742 specimens of the *An.* *gambiae* complex (between 109 to 240 per insecticide per site) were exposed to the WHO recommended diagnostic doses (2439 from Kedougou and 1303 from Wassadou-Badi). In both sites, a high number of mosquitoes were resistant to all five tested pyrethroids (mortality range 42.8–86.4%) as well as to the organochlorines (mortality range from 67.8–83% for dieldrin and 12.8–55.8% for DDT in Kedougou and Wassadou-Badi, respectively) (Fig. 2, Table 1). In the group of organophosphates, the populations of *An.* *gambiae* (*s.l*.) tested in both areas were susceptible to 5% malathion and 1% pirimiphos-methyl. Fenitrothion resistance (89% mortality rate, 95% CI: 85–95%) was detected in Kedougou, where *An.* *gambiae* (*s.l*.) populations were also resistant to bendiocarb 0.1% (Fig. 2).

**Table 1:** Mortality rates following insecticides (pyrethroids, DDT and dieldrin) exposure of *Anopheles* *gambiae* (*s.l*.) populations from Kedougou and Wassadou-Badi in October and November 2014

Locality	Insecticide	Mortality rate (%) (*n*)	KDT_50_ (min) (95% CI)	KDT_95_ (min) (95% CI)
Kedougou	DDT	12.8 (211)	161 (119.3–267.1)	784.95 (420.5–2287.9)
	Permethrin	53.2 (220)	164.89 (122.6–263.2)	1234.25 (634–3563.8)
	Deltamethrin	67.9 (240)	161 (128–222.8)	784.95 (490.7–1556.5)
	Lambda-cyhalothrin	57.1 (231)	53.92 (49.2–60.5)	149.27 (118.7–208.3)
	Cyfluthrin	81.4 (200)	22.72 (20.7–24.8)	65.63 (56.4–80.2)
	Alpha-cypermethrin	42.8 (217)	28.35 (26.8–30)	84 (74.9–96.6)
	dieldrin	67.8 (239)	97.56 (73.3–324.7)	223.3 (123.2–3120.3)
Wassadou-Badi	DDT	55.8 (116)	161 (119.3–267.1)	784.95 (420–2287.9)
	Permethrin	58 (119)	47.47 (43.4–52.9)	153.23 (121.9–210)
	Deltamethrin	68 (122)	58.8 (50.0–68.0)	113.38 (97.73–139.3)
	Lambda-cyhalothrin	53.4 (118)	63.56 (56.5–75.5)	174.65 (130.3–280.7)
	Cyfluthrin	86.4 (109)	41.7 (49.8–105.6)	87.45 (87.5–139.6)
	Alpha-cypermethrin	86 (110)	27.63 (25.6–29.7)	62.94 (55.8–73.6)
	dieldrin	83 (124)	0 (–)	0 (–)

*Abbreviations*: n, number of mosquitoes tested; 95% CI, 95% confidence interval; KDT_50_ and KDT_95_, knock down 50% and 95%; min, minutes

### Knockdown times/knockdown effect

In Kedougou, KDT_50_ greater than 60 min were recorded for DDT, permethrin and deltamethrin. In Wassadou-Badi a KDT_50_ greater than 60 min were noted with DDT and lambda-cyhalothrin. The KDT_50_ value for permethrin was 3.5 times higher in Kedougou compared to Wassadou-Badi (*χ*^2^ = 10.029, *df* = 1, *P* = 0.0015). However, KDT_50_ value for deltamethrin in Kedougou was 2.7 higher than KDT_50_ value of Wassadou-Badi (*χ*^2^ = 3.0083, *df* = 1, *P* = 0.0828) and no significant difference was observed between these two sites. Conversely, for cyfluthrin and lambda-cyhalothrin, KDT_50_ were respectively 1.8 and 1.18 times higher in Wassadou-Badi (*χ*^2^ = 19.3177, *df* = 1, *P* < 0.0001; *χ*^2^ = 15.2239, *df* = 1, *P* < 0.0001). Cyfluthrin and alpha-cypermethrin had the lowest KDT50 compared to other pyrethroids tested (Table 1).

### *Vgsc*-1014F, *Vgsc*-1014S, ace-1 (G119S), rdl-A296S and *rdl*-A296G mutation frequencies in *An. arabiensis*, *An. coluzzii* and *An. gambiae* (*s.s.*)

The frequency of *kdr* (*Vgsc*) gene mutations was different among the three different members of the *An.* *gambiae* complex. The wild-type allele dominated in both Kedougou and Wassadou-Badi in *An.* *arabiensis*. In *An.* *gambiae* (*s.s*.) and *An.* *coluzzii* population, a predominance of FF homozygotes was noted in both sites for the *Vgsc*-1014F mutation. The results revealed two homozygous hybrids resistant to the *Vgsc*-1014F mutation. The *Vgsc*-1014S mutation was not found any member of the *An*. *gambaie* complex in Kedougou but was predominant in the *An.* *arabienesis* in Wassadou-Badi.

The allelic frequencies of the *Vgsc*-1014F mutation (Kedougou: Fisher’s exact test: OR: 221.48, 95% CI: 29.3–9494.2, *P* < 0.001; Wassadou-Badi: *χ*^2^ = 455.3289, *df* = 2, *P* < 0.001) and *Vgsc*-1014S (Wassadou-Badi: Fisher’s exact test: OR: 0.00, 95% CI: 0.00–0.96, *P* < 0.001) were significantly higher in *An.* *gambiae* (*s.s.*) compared to *An.* *coluzzii* and *An.* *arabiensis* (Table 2).

**Table 2:** Genotypes and allelic frequencies of mutations *Vgsc*-1014F, *Vgsc*-1014S, *Ace-1* (*G119S*), *rdl*-A296S, and *rdl*-A296G in *An.* *arabiensis*, *An.* *coluzzii* and *An.* *gambiae* (*s.s.*) in Kedougou and Wassadou-Badi in October and November 2014

Localities	Species	*Vgsc*-1014F				*P*	*Vgsc-*1014S				*P*
		LL	LF	FF	(freq R)		LL	LS	SS	(freq R)	
Kedougou	*An.* *arabiensis*	23	1	3	0.129	˂ 0.001	14	0	0	0.00	na
	*An.* *coluzzii*	0	1	18	0.973		0	0	0	0.00	
	*An.* *gambiae* (*s.s.*)	0	1	298	0.998		2	0	0	0.00	
Wassadou-Badi	*An.* *arabiensis*	114	3	3	0.037	< 0.001	55	11	12	0.22	< 0.001
	*An.* *coluzzii*	10	5	10	0.500		8	0	0	0.00	
	*An.* *gambiae* (*s.s.*)	3	0	134	0.978		0	0	10	1	

Localities	Species	*Ace-1* (G119S)				*P*	*Rdl*-A296S or *Rdl-*A296G				*P*
		GG	GS	SS	(freq R)		AA	AG	GG	(freq R)	
Kedougou	*An.* *arabiensis*	1	0	0	0.00	0.33	25	1	0	0.019	0.014
	*An.* *coluzzii*	19	1	0	0.025		11	1	0	0.041	
	*An.* *gambiae* (*s.s.*)	101	28	7	0.154		145	49	2	0.135	
Wassadou-Badi	*An.* *arabiensis*	38	3	0	0.036	0.043	32	0	0	0.00	0.124
	*An.* *coluzzii*	21	0	0	0 .00		4	0	0	0.00	
	*An.* *gambiae* (*s.s.*)	57	11	6	0.155		44	4	1	0.061	

*Abbreviations:* *P*, probability of significant difference for each mutation among species within each site; L, leucine; F, phenylalanine; S, serine; G, glycine; A, alanine; Freq R, frequency of resistant allele; na, not applicable, FF, phenyl alanine- phenyl alanine

The wild-type allele was the most frequent allele for the *ace-1*^*R*^ for all species of the *An.* *gambiae* complex in both sites. The frequency of the *ace-1*^*R*^ (G119S) mutation was low in both sites and heterozygotes genotypes (GS) were predominant for carriers of an 119S allele.

In Wassadou-Badi, a relatively higher allelic frequency was noted in *An.* *gambiae* (*s.s*.), the only species in which all SS homozygotes were found (Table 2). As with a*ce-1*^*R*^, the predominant allele for *rdl* gene was the wild type allele. The mean allelic frequencies of A296S or A296G were significantly different among species of the *An.* *gambiae* complex in Kedougou (Fisher’s exact test: OR: 7.95, 95% CI: 1.30–326.6, *P* = 0.0147), but not in Wassadou-Badi (Fisher’s exact test: OR: inf, *P* = 0.12). However, only *An.* *gambiae* (*s.s.*) population has homozygous (GG) for A296G *rdl* allele (Table 2).

### Allelic frequencies at the *Vgsc*-1014F, *Vgsc*-1014S, ace-1^R^ (G119S) and *rdl*-A296S or *rdl*-A296G locus according to the phenotype after insecticide exposure

Table 3 shows the allelic frequencies of the *Vgsc*-1014F, *Vgsc*-1014S, G119S and *rdl-*A296G or *rdl-*A296S mutations in the selected specimens that survived or died after exposure to insecticides.

**Table 3:** Numbers of specimens and frequencies of G119S and *rdl* A296G or *rdl* A296S mutations by surviving or dead phenotypes in *An.* *arabiensis*, *An.coluzzii* and *An.* *gambiae* (*s.s.*) of Kedougou and Wassadou-Badi in October and November 2014

Locality	Phenotype	*n*	*An.* *arabiensis* (freq R)	*P*	*n*	*An.* *coluzzii* (freq R)	*P*	*n*	*An.* *gambiae* (*s.s.*) (freq R)	*P*
Kedougou										
*Vgsc-*1014F	Surviving	11	0.31	0.031	18	0.97		248	1.0	
	Dead	16	0.06		0	0.0	na	15	0.97	0.057
*Vgsc*-1014S	Surviving	2	0.00	na	1	0.00		42	0.00	
	Dead	12	0.00		0	0.00		16	0.00	na
Wassadou-Badi										
*Vgsc*-1014F	Surviving	51	0.068	0.017	11	0.6	0.44	100	0.98	
	Dead	41	0		8	0.37		9	0.88	0.07
*Vgsc*-1014S	Surviving	24	0.48		3	0.00		51	1.0	
	Dead	33	0.07	< 0.001	7	0.00	na	6	0.00	na
Kedougou										
*Ace-1* G119S	Surviving	21	0.00	na	0	0.00		31	0.47	
	Dead	33	0.00		2	0.00	na	22	0.11	< 0.001
*Rdl*-A296S or *Rdl*-A296G	Surviving	2	0.00	na	2	0.00		34	0.21	
	Dead	0	0.00		0	0.00	na	15	0.00	0.004
Wassadou-Badi										
*Ace-1* G119S	Surviving	0	0.00	na	1	0.00	na	11	0.36	0.034
	Dead	11	0.00		1	0.00		6	0.00	
*Rdl*-296S or *Rdl-*A296G	Surviving	2	0.00	na	–	–	–	–	–	–
	Dead	6	0.00		–	–	–	–	–	–

*Abbreviations*: freq R, allelic frequency of mutation studied; *n*, number of treated specimens; na, not applicable

In both study areas, *An.* *gambiae* (*s.s.*) was the predominant species among surviving specimens (96.6% in Kedougou; 64.1% in Wassadou-Badi). The percentage of *An.* *gambiae* (*s.s.*) was higher in surviving compared to the dead specimens (*χ*^2^ = 32.4, *df* = 1, *P* < 0.0001) while *An.* *arabiensis* (82.7%, *n* = 52) predominated only in dead specimens in Wassadou-Badi.

In *An.* *gambiae* (*s.s*.), the frequencies of resistant allele in surviving *versus* dead specimens after exposition to DDT and pyrethroids were comparable for the 1014F allele (Fisher’s exact test: OR: 0.00; 95% CI: 0.0–2.4, *P* ≥ 0.057) and significantly different between those specimens exposed to bendiocarb and fenitrothion for the *ace-l*^*R*^ (G119S) allele (Fisher’s exact test: OR: 0.15; 95% CI: 0.040–0.45, *P* ≤ 0.001) in both sites (Table 3).

In Wassadou-Badi, the frequencies of the 1014S allele in *An.* *arabiensis* (0.34 *vs* 0.06, Fisher’s exact test: OR: 0.09; 95% CI: 0.02–0.28, *P* < 0.001) as well as that of the *rdl*-296G allele in *An.* *gambiae* (*s.s*.) (0.21 *vs* 0.0, Fisher’s exact test: OR: 0.00; 95% CI: 0.00–0.60, *P* = 0.004) were higher in surviving compared to the dead specimens after exposure to dieldrin in Kedougou. On the other hand, there was no significant difference between the frequencies of the 1014F allele in dead and surviving specimens in both *An.* *gambiae* (*s.s.*) (0.98 *vs* 0.88, Fisher’s exact test, OR: 0.17; 95% CI: 0.02–1.96, *P* = 0.07) and *An.* *arabiensis* (0.068 *vs* 0.0, Fisher’s exact test: OR: 0.00; 95% CI: 0.0–0.0.83, *P* = 0.017).

## Discussion

This study aimed to update data relating to insecticide susceptibility and to determine the frequencies of mutations of *kdr* (*Vgsc*-1014F and *Vgsc*-1014S), *ace-1*^*R*^ and *rdl* alleles associated with the resistance of *An.* *gambiae* (*s.l*.) populations to insecticides in southeastern Senegal.

The results of the WHO susceptibility tests showed vector resistance to pyrethroids organochlorines (DDT and dieldrin) and carbamates insecticides that are recommended by PQT-VC (Prequalification Team: Vector Control Products). These insecticides are the only ones currently approved for LLIN treatment [34, 35], and are offered by nongovernmental organizations such as the United States President’s Malaria Initiative (PMI) and Senegal River Basin Development Organization (OMVS). The use of LLINs over several years could have led to the increase of resistance genes in vectors of *An.* *gambiae* species complex, through selection pressure [36, 37]. The resistance of *An.* *gambiae* (*s.l*.) to pyrethroids has been shown to be strongly associated with their excessive use in agriculture especially in cotton growing areas [38].

Moreover, *An.* *gambiae* (*s.l*.) populations in the area were also resistant to organochlorines (DDT and dieldrin). Since the first malaria eradication attempt, DDT and dieldrin resistance phenotypes have been reported in many African countries by Hamon & Garrett-Jones [39]. Despite several decades of non-use, DDT may persist in the environment due to lack of microbial degradation system [40].

Previous studies have reported resistance only to DDT and pyrethroids in southeastern and central Senegal where LLIN use is high [19, 20]. However, unlike previous studies conducted in Senegal, this study shows that vectors are resistant to almost all tested pyrethroids and bendiocarb.

Bioassays likewise showed resistance to bendiocarb in Kedougou. This resistance could result from selection pressure in larval from insecticide residues (bendiocarb) used on cotton crops by SODEFITEX [41]. This phenotypic resistance to bendiocarb should be closely monitored as there is cross-resistance to carbamates and organophosphates.

The search for mutations involved in the phenotypic resistance of *An.* *gambiae* (*s.l*.) population to insecticides showed the presence of *Vgsc*-1014F, *Vgsc*-1014S, *ace-1* (G119S) and *rdl*-A296S or *rdl*-A296G mutations. The *Vgsc*-1014S mutation was not found in *An.* *gambiae* (*s.l*.) from Kedougou, where the *Vgsc*-1014F was at 0.99 *in* *An.* *gambiae* (*s.s*)*.* Although not yet fixed in Wassadou-Badi, the allelic frequency of *Vgsc*-1014F mutation was greater than 0.50.

The frequency of the *Vgsc*-1014F mutation was higher both in the surviving and dead phenotypes in the *An.* *gambiae* (*s.s.*) populations from Kedougou. The frequency of the *Vgsc*-1014S mutation in *An.* *arabiensis* populations from Wassadou-Badi was higher in the surviving than the dead specimens whereas no correlations were detected between the *Vgsc*-1014F mutation and the resistance phenotype in *An.* *gambiae* (*s.s.*) and *An.* *coluzzii*. It is therefore likely that mechanisms other than *Vgsc*-1014F mutation are involved in the insecticide resistance of these species. This hypothesis should be investigated in the future. These results are in line with those of Thiaw et al*.* [20] and Ahoua et al*.* [42], who found no correlation between the *kdr* mutation and the phenotypic alive or dead phenotypic, respectively, in *An.* *arabiensis* and *An.* *coluzzii*. Only the *Vgsc*-1014F mutation was noted in *An.* *coluzzii*. This finding could be explained by introgression from *An.* *gambiae* (*s.s.*) to *An.* *coluzzii* [43, 44]. Furthermore, our results show an absence of the *Vgsc*-1014S mutation in *An.* *coluzzii*. This finding is similar to results obtained in Benin [45], but not those obtained in Cameroon [46] and in the Republic of Equatorial Guinea [47]. The occurrence of the *Vgsc*-1014F mutation was detected in two hybrids (*An.* *gambiae* (*s.s.*)/*An.* *coluzzii*) and were homozygote-resistant genotype (FF). To our knowledge, this is the first report of this mutation in hybrids of *An.* *gambiae* (*s.s.*) and *An.* *coluzzii* in Senegal. Other mutations could be involved in resistance of *An.* *gambiae* (*s.l*.) to insecticides, including the *Vgsc*-1575Y mutation [48] that was not investigated in this study.

With a significantly higher frequency in surviving specimens after exposure, the study shows that the *ace-1*^*R*^ mutation was implicated in phenotypic resistance of *An.* *gambiae* (*s.s.*) to bendiocarb. The involvement of the *ace-1*^*R*^ mutation in the phenotypic resistance to bendiocarb has been reported in *An.* *gambiae* (*s.s.*) populations from Côte Ivoire [42] and Ghana [49]. However, it was not present in surviving *An.* *arabienesis.*

The presence of heterozygotes in surviving specimens may explain the resistance of *An.* *gambiae* (*s.l*.) population to carbamates (bendiocarb) from Kedougou and Wassadou-Badi and organophosphates (fenitrothion) from Kedougou area.

Often associated with *rdl* mutation (*rdl*-A246S or *rdl*-A296G), the phenotypic resistance to dieldrin was found in *An.* *gambiae* (*s.l*.) populations in both localities. A similar result was obtained in Benin [50]. The allelic frequencies obtained in our study are quite similar to those described by Corbel et al. [50]. The phenotypic resistance to dieldrin could be explained by the long use of dieldrin in the past or other insecticides belonging to different families (such as fipronil or lindane) with the same mode of action as dieldrin on one hand and by the presence of *rdl-* A296G mutation, which is associated with a 2La chromosomal polymorphic on the other hand [50, 51]. This is a very stable polymorphic inversion that limits crossover and would help preserve this mutation in a given population. The occurrence of multiple-resistance locus in *An.* *gambiae* (*s.s*), the main malaria vector in the study area, is indicative of the genes involved in resistance to the insecticides used in this area.

## Conclusions

The study demonstrates phenotypic resistance in *An.* *gambiae* (*s.l*.) population to DDT, pyrethroids, benbiocarb and fenitrothion in southeastern Senegal. The relatively higher frequency in specimens surviving insecticide exposure demonstrates the role of target site modifications, including *Vgsc*-1014F and *Vgsc*-1014S, *ace-1*^*R*^ and *rdl*-A296S or *rdl*-A296G. Though they are one of the main factors, investigation of other mechanisms involved remains necessary for better management of the resistance in *An.* *gambiae* (*s.l*.) populations. Resistance to insecticides may jeopardize the effectiveness of the main strategies (indoor residual spraying (IRS) of persistent insecticides and the use LLIN mosquito nets) to reduce malaria transmission in the area.

## Data Availability

Data supporting the conclusions of this article are included within the article. The data used and analyzed during the current study are available from the corresponding author upon reasonable request.

## Abbreviations

CTAB: Cetyl trimethyl ammonium bromide
PCR: Polymerase chain reaction
*kdr*: Knockdown resistance
*rdl*: Resistance to dieldrin
OMVS: Senegal River Basin Development Organization
IMP-PCR: Intentional mismatches primer-PCR
DDT: Dichloro diphenyltrichloroethane
*ace-1*: Target-site resistance gene for carbamate and organophosphate insecticides conferring insensitive acetyl cholinesterase
*Vgsc*: Voltage-gated sodium channel
WHO: World Health Organization
LLINs: Long-lasting insecticide-treated nets
*ace1*^*R*^: Acetyl cholinesterase insensible (G119S)
*Vgsc*-1575Y: Polymorphism non-synonym
PMI: United States President’s Malaria Initiative
KDT_50_ and KDT_95_: Knockdown 50% and 95%
GABA: Gamma-amino butyric acid
IRS: Indoor residual spraying of insecticides
PQT-VC: Prequalification team-vector control products
*s.l.*: *sensu* *lato*
*s.s.*: *sensu* *stricto*
P: Probability

## References

[1] WHO. World malaria report 2019. Geneva: World Health Organization; 2019. https://www.who.int/publications/i/item/world-malaria-report-2019.

[2] WHO. World malaria report 2017. Geneva: World Health Organization; 2017. https://www.who.int/publications/i/item/world-malaria-report-2017.

[3] Ranson H, N’Guessan R, Lines J, Moiroux N, Nkuni Z, Corbel V (2011). Pyrethroid resistance in African anopheline mosquitoes: what are the implications for malaria control?. Trends Parasitol.

[4] Dabiré RK, Namountougou M, Diabaté A, Soma DD, Bado J, Toé HK (2014). Distribution and frequency of kdr mutations within *Anopheles**gambiae* (*s.l.*) populations and first report of the ace.1 G119S mutation in *Anopheles**arabiensis* from Burkina Faso (West Africa). PLoS ONE.

[5] Sangba MLO, Deketramete T, Wango SP, Kazanji M, Akogbeto M, Ndiath MO (2016). Insecticide resistance status of the *Anopheles**funestus* population in Central African Republic: a challenge in the war. Parasit Vectors.

[6] Camara S, Koffi AA, Alou LP, Koffi K, Kabran JPK, Koné A (2018). Mapping insecticide resistance in *Anopheles**gambiae* (*s.l.*) from Côte d’Ivoire. Parasit Vectors.

[7] Fossog Tene B, Poupardin R, Costantini C, Awono-Ambene P, Wondji CS, Ranson H (2013). Resistance to DDT in an urban setting: common mechanisms implicated in both M and S forms of *Anopheles gambiae* in the city of Yaoundé Cameroon. PLoS ONE.

[8] Awolola TS, Oduola OA, Strode C, Koekemoer LL (2008). Evidence of multiple pyrethrinoid resistance mechanism in malaria vector *Anopheles gambiae* (*s.s*) from Nigeria. Am J Trop Med Hyg.

[9] Wondji CS, Coleman M, Kleinschmidt I, Mzilahowa T, Irving H, Ndula M (2012). Impact of pyrethroid resistance on operational malaria control in Malawi. Proc Natl Acad Sci USA.

[10] Foster GM, Coleman M, Thomsen E, Ranson H, Yangalbé-Kalnone E, Moundai T (2016). Spatial and temporal trends in insecticide resistance among malaria vectors in Chad highlight the importance of continual monitoring. PLoS ONE.

[11] Keita K, Camara D, Barry Y, Osse R, Wang L, Sylla M (2017). Species identification and resistance status of *Anopheles**gambiae* (s.l.) (Diptera: Culicidae) mosquitoes in Guinea. J Med Entomol.

[12] Okorie PN, Ademowo GO, Irving H, Kelly-Hope LA, Wondji CS (2015). Insecticide susceptibility of *Anopheles coluzzii* and *Anopheles gambiae* mosquitoes in Ibadan, South-West Nigeria. Med Vet Entomol.

[13] Martinez-Torres D, Chandre F, Williamson MS, Darriet F, Berge JB, Devonshire AL (1998). Molecular characterization of pyrethroid knockdown resistance (*kdr*) in the major malaria vector *Anopheles gambiae s.s*. Insect Mol Biol.

[14] Ranson H, Jensen B, Vulule JM, Wang X, Hemingway J, Collins FH (2000). Identification of a point mutation in the voltage-gated sodium channel gene of Kenyan *Anopheles gambiae* associated with resistance to DDT and pyrethroids. Insect Mol Biol.

[15] Brooke BD, Hunt RH, Coetzee M (2000). Resistance to dieldrin + fipronil assorts with chromosome inversion 2La in the malaria vector *Anopheles gambiae*. Med Vet Entomol.

[16] Weill M, Lutfalla G, Mogensen K, Chandre F, Berthomieu A, Berticat C (2003). Comparative genomics: insecticide resistance in mosquito vectors. Nature.

[17] Du W, Awolola TS, Howell P, Koekemoer LL, Brooke BD, Benedict MQ (2005). Independent mutations in the Rdl locus confer dieldrin resistance to *Anopheles gambiae* and *An.**arabiensis*. Insect Mol Biol.

[18] Faye O, Konate L, Diop A. Profil entomologique du paludisme au Sénégal. Unpublished: Ministère de la Santé et de la Prévention Médicale; 2011.

[19] Niang EA, Konaté L, Diallo M, Faye O, Dia I (2016). Patterns of insecticide resistance and knock down resistance (kdr) in malaria vectors *An. arabiensis*, *An. coluzzii *and *An. gambiae* from sympatric areas in Senegal. Parasit Vectors.

[20] Thiaw O, Doucouré S, Sougoufara S, Bouganali C, Konaté L, Diagne N (2018). Investigating insecticide resistance and knock-down resistance (*kdr*) mutation in Dielmo, Senegal, an area under long lasting insecticidal-treated nets universal coverage for 10 years. Malar J.

[21] Food and Agriculture Organization. Animal Production and Health paper 41, Integrating crops and livestock in West Africa. 1983. https://www.fao.org/docrep/004/x6543e/x6543e01.htm. Accessed 25 Mar 2014.

[22] Bulletin épidémiologique annuel du paludisme au SENEGAL; 2016. https://www.pnlp.sn/2016.

[23] Bulletin épidémiologique annuel du paludisme au SENEGAL; 2014. https://www.pnlp.sn/2014/.

[24] Dia I, Diop T, Rakotoarivony I, Kengne P, Fontenille D (2003). Bionomics of *Anopheles gambiae* Giles, *An. arabiensis *Patton, *An. funestus* Giles and *An. nili* (Theobald) (Diptera: Culicidae) and transmission of *Plasmodium falciparum* in a Sudano-Guinean zone (Ngari, Senegal). J Med Entomol.

[25] Ndiath MO, Mazenot C, Gaye A, Konate L, Bouganali C, Faye O (2011). Methods to collect *Anopheles* mosquitoes and evaluate malaria transmission: a comparative study in two villages in Senegal. Malar J.

[26] WHO. Test procedures for insecticide resistance monitoring in malaria vectors mosquitoes. Geneva: World Health Organization; 2013. https://www.who.int/malaria/publications/atoz/9789241505154.

[27] Abbott WS (1925). A method of computing the effectiveness of an insecticide. J Ecol Entomol.

[28] Gillies MT, De Meillon B (1968). The *Anophelinae* of Africa South of the Sahara (Ethiopian zoogeographical region). Publ S Afr Inst Med Res.

[29] Murray MG, Thompson WF (1980). Rapid isolation of high molecular weight plant DNA. Nucleic Acids Res.

[30] Wilkins EE, Howell PI, Benedict MQ (2006). IMP PCR primers detect single nucleotide polymorphisms for *Anopheles gambiae* species identification, Mopti and Savanna rDNA types, and resistance to dieldrin in *Anopheles arabiensis*. Malar J.

[31] Methods in *Anopheles* research manual full version (MR4). 2014. p. 250–1. https://www.beiresources.org.

[32] Weill H, Hughes JM, Churg AM (2004). Changing trends in US mesothelioma incidence. Occup Environ Med.

[33] R Development Core Team. R: a language and environment for statistical computing. Vienna: R Foundation for Statistical Computing. 2014. https://www.R-project.org/.

[34] Hakizimana E, Karema C, Munyakanage D, Iranzi G, Githure J, Tongren JE (2016). Susceptibility of *Anopheles gambiae* to insecticides used for malaria vector control in Rwanda. Malar J.

[35] Dadzie S, Appalu MA, Kerah-Hinzoumbé C, Akogbeto MC, Adimazoya M, Israël DK (2016). Species composition and insecticide resistance status of *Anopheles**gambiae* (*s.l.*) (Culicidae) in Kome, southern Chad and the implications for malaria control. Parasit Vectors.

[36] Zoh DD, Ahoua Alou LP, Toure M, Pennetier C, Camara S, Traore DF (2018). The current insecticide resistance status of *Anopheles gambiae* (*s.l.*) (Culicidae) in rural and urban areas of Bouaké, Côte d’Ivoire. Parasit Vectors.

[37] Thwing JI, Perry RT, Townes DA, Diouf MB, Ndiaye S, Thior M (2011). Success of Senegal’s first nationwide distribution of long-lasting insecticide-treated nets to children under five - contribution toward universal coverage. Malar J.

[38] Diabate A, Baldet T, Fabrice C, Akogbeto M, Guiguemde TR, Darriet F (2002). the role of agricultural use of insecticides in resistance to pyrethroids in *Anopheles**gambiae**s.l.* in Burkina Faso. Am J Trop Med Hyg.

[39] Hamon J, Garrett-Jones C (1963). La résistance aux insecticides chez des vecteurs majeurs du paludisme et son importance opérationnelle. Bull OMS.

[40] Samir SR, Leo MLN (2012). Pesticides: evaluation of environmental pollution.

[41] Appel d’offre N°39/2014/DOC/DPC portant sur la fourniture de produits insecticides destines à la cotonnière. https://www.sodefitex.sn/images/2014.pdf.

[42] Ahoua AL, Koffi AA, Adja MA, Assi SB, Kouassi PK, N’Guessan R (2012). Status of pyrethroid resistance in *Anopheles**gambiae**s.s.* M form prior to the scaling up of long-lasting insecticidal nets (LLINs) in Adzopé, eastern Côte d’Ivoire. Parasit Vectors.

[43] Weill M, Chandre F, Cécile B, Manguin S, Akogbeto M, Pasteur N (2000). The *kdr* mutation occurs in the mopti form of *Anopheles gambiae s.s.* through introgression. Insect Mol Biol.

[44] Diabate A, Brengues C, Baldet T, Dabire KR, Hougard JM, Akogbeto M (2004). The spread of the Leu-Phe *kdr* mutation through *Anopheles gambiae* complex in Burkina Faso: genetic introgression and *de novo* phenomena. Trop Med Int Health.

[45] Djègbè I, Boussari O, Sidick A, Martin T, Ranson H, Chandre F (2011). Dynamics of insecticide resistance in malaria vectors in Benin: first evidence of the presence of L1014S *kdr* mutation in *Anopheles gambiae* from West Africa. Malar J.

[46] Reimer L, Fondjo E, Patchoké S, Diallo B, Lee Y, Ng A (2008). Relationship between *kdr* mutation and resistance to pyrethroid and DDT insecticides in natural populations of *Anopheles gambiae*. J Med Entomol.

[47] Ridl FC, Bass C, Torrez M, Govender D, Ramdeen V, Yellot L (2008). A pre-intervention study of malaria vector abundance in Rio Muni, Equatorial Guinea: their role in malaria transmission and the incidence of insecticide resistance alleles. Malar J.

[48] Jones CM, Liyanapathirana M, Agossa FR, Weetman D, Ranson H, Donnelly MJ (2012). Footprints of positive selection associated with a mutation (N1575Y) in the voltage-gated sodium channel of *Anopheles gambiae*. Proc Natl Acad Sci USA.

[49] Essandoh J, Yawson AE, Weetman D (2013). Acetylcholinesterase (Ace-1) target site mutation 119S is strongly diagnostic of carbamate and organophosphate resistance in *Anopheles gambiae**s.s.* and *Anopheles coluzzii* across southern Ghana. Malar J.

[50] Corbel V, N’Guessan R, Brengues C, Chandre F, Djogbenou L, Martin T (2007). Multiple insecticide resistance mechanisms in A*nopheles gambiae* and *Culex quinquefasciatus* from Benin, West Africa. Act Trop.

[51] Wondji CS, Dabire R, Tukur Z, Irving H, Djouaka R, Djouaka J (2011). Identification and distribution of a GABA receptor mutation conferring dieldrin resistance in the malaria vector *Anopheles**funestus* in Africa. Insect Biochem Mol Biol.

